# Authors’ Response to Peer Reviews of “Why We Are Losing the War Against COVID-19 on the Data Front and How to Reverse the Situation”

**DOI:** 10.2196/29421

**Published:** 2021-05-05

**Authors:** David Prieto-Merino, Rui Bebiano Da Providencia E Costa, Jorge Bacallao Gallestey, Reecha Sofat, Sheng-Chia Chung, Henry Potts

**Affiliations:** 1 Faculty of Epidemiology & Population Health London School of Hygiene & Tropical Medicine London United Kingdom; 2 Applied Statistical Methods in Medical Research Group Catholic University of San Antonio in Murcia Murcia Spain; 3 Institute of Health Informatics University College London London United Kingdom; 4 University of Medical Sciences Havana Cuba

**Keywords:** COVID-19, learning health systems

*This is the authors’ response to peer-review reports for the paper “Why We Are Losing the War Against COVID-19 on the Data Front and How to Reverse the Situation”.*

## Round 1 Review

The authors of the manuscript [1] thank the reviewers [2-5] for their comments. In addition to making changes in response, we have also updated the paper in various places given the changing nature of the pandemic and the response to it.

### Reviewer A

#### General Comments

The *What Can Be Done?* section has been rewritten with much more detail.

#### Specific Comments

1. Thank you for your suggestion. Details on procedures for data collection and processing will be very dependent on the characteristics of each health system and the data routinely collected for clinical care, so it will be difficult for us to suggest here a particular system. However, in point 2 of the *What Can Be Done?* section, we have added some points that any health system should consider when planning the data collection strategy.

2. Thank you for the suggestion; we actually asked some expert colleagues informally for their opinion on a possible data structure, and we received different answers (more or less overlapping in some variables). We realized that defining the data set is a task that should be done by an expert panel considering not only the disease but also the characteristics of the health system that will collect the data and the cost/benefit of collecting each piece of data. We have now elaborated on this idea in point 1 in the *What Can Be Done?* section.

3. “Immediately used for data collection” is a relative term as any platform would have to be adapted to the decided data structure. There are many tools out there with different degrees of adaptability, but the point here is to use as much as possible the existing health information systems in place to extract the data and create feedback loops to the health care system, rather than setting up separate platforms.

4. Automatic data processing is not a problem of software. Any modern statistical software can be programmed to repeat data processing of analytical routines. The key issue is to decide what to analyze and with what statistical models. The statistical experts will define the models needed to answer the questions asked by the medical/epidemiological experts.

### Reviewer L

#### Specific Comments

##### Major Comments

1. Thank you for the suggestion; we have tried to improve this, including adding some more citations.

2. Thank you for highlighting these interesting points. We have internally discussed the idea of *who* should be coordinating the international effort to combine data and analysis and we largely agree with it. However, we feel that exceeds the scope of our original aim for this paper—to highlight the missing opportunity of implementing learning health system within health care systems and taking it to the next level of international coordination of health learning systems.

About the funding, that will depend very much on the kind of health systems and even the society where the health system is embedded, and we cannot comment on each case separately. For example, in the countries where the authors work and live, the health systems are mainly publicly funded, and we would expect the competent public authorities to make that funding effort just as they did with the extra effort for medical care during the pandemic. Other systems might have to find other resources. In any case, one needs to consider this as an investment because the earlier we learn to effectively manage the pandemic, the more we can reduce costs in the long run.

3. We do not really know the answer to this certainly very important question, but we believe it is complex enough to require a separate piece of research. For the problem that we are trying to address here, about implementing health learning systems, we must assume that each system will try to find the most up-to-date and “reliable” scientific information available at the time. We understand that the most recent information could be incomplete and possibly unreplicated so far. So, the learning health system should be constantly revised and updated as new solid information is made available. We have added a new point 8 in the *What Can Be Done?* section discussing this issue.

### Anonymous [4]

1. Thank you for the comment; we certainly do not try to predict how information on the pandemic will evolve in the future. We rather try to denounce how the opportunity has been missed to implement a useful, responsive health information system embedded in the health care system.

2. Thank you for the comment. Because the paper is not a typical research paper with an introduction, methods, results, discussion, etc, we found it difficult to follow a similar structure in the abstract. However, we have revised the abstract to clarify the purpose of the research in its final paragraph.

3. Thank you for the comment. We do not have an *Introduction* section as such, but we have split this into 2 sections, *The Problem* and *What Do We Need to Solve the Problem?*, which we believe address the purpose of this paper.

4. Because the paper is not a typical research paper, we do not have an *Introduction* section as such.

5. Thank you for the references. They look very relevant for a discussion on public policies to tackle the pandemic. We are not sure how to fit them in our paper, which focuses on learning health systems, but we will consider them in future works.

6. Thank you for the comment. Because the paper is not a typical research paper, we found it difficult to follow a typical structure in the *Conclusion* section. However, we have added some sentences at the beginning and a final sentence to address your points.

### Anonymous [5]

#### Specific Comments

##### Major Comments

1. Thank you for the suggestion. We actually asked some expert colleagues informally for their opinion on a possible data structure, and we received different answers (more or less overlapping in some variables). We realized that defining the data set is a task that should be done by an expert panel considering not only the disease but also the characteristics of the health system that will collect the data and the cost/benefit of collecting each piece of data. We have now elaborated on this idea in point 1 of the *What Can Be Done?* section.

2. Thanks for the comment. In the section *What Is Being Done?*, we did include references to some initiatives that were taken to collect and analyze COVID-19 clinical data. There are, of course, many more, but it is not the aim of this paper to review them. We included some points discussing the shortcomings of many of these systems, mostly independent from actual health care systems. We have now included references to some initiatives at the national level (most notoriously one in the United Kingdom) but note that we still fall short of the concept of the learning health system that we propose.

3. Yes, we can see your point, although we regard electronic medical record (EMR) vendors as service providers who do not actually own the data or the right to exploit it. We believe this to be the situation at least in Europe where the data mostly “belong” to the institutions providing the health services (in the sense that they are the “data controllers” under GDPR [General Data Protection Regulation] regulations). We are assuming that is up to these institutions to decide what data they collect, process, analyze, and share, and we view EMR vendors just as companies providing software support. Maybe in other countries the situation is different. To discuss all these different situations will make the paper too long and probably deviate from the main message of implementing learning health systems integrated with health care systems. Maybe one can consider EMR vendors as an element of the “health care system” in general with a role to play in implementing the learning health systems.

## Round 2

### Reviewer L

#### Specific Comments

1. Thank you for your comment. We acknowledge that more references could have been provided. On the other hand, we would not want to overload the reader with references for points that are quite well known to the public, such as the situation of the pandemic and the actions of many countries to tackle it, that we mention in the first paragraph. Regarding point 7, this proposition is of a speculative nature rather than affirmative. We have not verified it with data; we have just derived it from the logic that the more appealing a given resource is, the more demanded it will be by the potential beneficiaries of its use and the more likely that a bottleneck will be created if the access points to that resource are kept at a constant capacity of delivery. Certainly, these bottlenecks would be ameliorated if the capacity to deliver that resource is improved to keep up with the demand, and we have included this idea by adding the following sentence “(unless more resources are put into place to deal with the requests).”

2. In the third paragraph of *The Problem*, we mention some limitations of the epidemiological data. Epidemiological data are very valuable for monitoring the epidemic and deciding on public health measures but are more limited for understanding the risk factors for getting infected and for predicting the prognosis of patients or their response to treatment. Epidemiological data might provide the distribution of cases by age groups, gender, ethnicity, etc, but they provide limited information on the combinations of these risk factors. If we want to combine the distribution of several risk factors (eg, sex, age, ethnicity, socioeconomic status, previous comorbidities, etc), it is unlikely that the epidemiological reports will provide this. Another limitation is that continuous variables such as age, BMI, blood pressure, and respiratory rate are categorized to produce report tables, and the usefulness of the variable is reduced. Also, epidemiological reports normally do not include parameters collected in hospitals during the progression of the disease that could be critical for creating prognostic models to predict which patients would benefit more from what treatments. We have included some sentences clarifying these limitations in that third paragraph.

3. A critical point to solve a research question with a learning health system is to find suitable cases. The learning health system would check every new patient in the system to see if they match the research question criteria to be included. A health system implemented only at one center will answer the question with enough certainty when enough patients have been recruited at that center. Noncoordinated health systems implemented separately at different centers will take as much time to get to an answer, but if they were coordinated and share data pertaining to new, valid patients, then they will be closer to answering the question.

4. Our point is precisely that at the moment current practices do not implement what we call a health system as defined in our references 3 and 4. We have tried to illustrate in the section *The Problem* the failure to answer the most pressing clinical questions even after having millions of cases, hundreds of thousands of deaths, and vast amounts of epidemiological data. This failure is in itself a manifestation of the lack of a health learning system (we are not learning fast enough). In section *What Can Be Done?*, we provide some examples of the innovative initiatives taken to tackle this problem, but we also argue why they fall short of being a health learning system. In this section, we propose the characteristics of what we would consider a learning health system, which can be summarized as:

1. An organizational architecture that facilitates the formation of communities of patients, families, front-line clinicians, researchers, and health system leaders who collaborate to produce and use big data;
2. Large electronic health and health care data sets (big data);
3. Quality improvement for each patient at the point of care brought about by the integration of relevant new knowledge generated through research;
4. Observational research and clinical trials done in routine clinical care settings.

## Abbreviations

EMR: electronic medical record
GDPR: General Data Protection Regulation

## References

[1] Prieto-Merino D, Bebiano Da Providencia E Costa R, Bacallao Gallestey J, Sofat R, Chung S-C, Potts H (2021). Why We Are Losing the War Against COVID-19 on the Data Front and How to Reverse the Situation. JMIRx Med.

[2] Lafon-Hughes L (2021). Peer Review of "Why We Are Losing the War Against COVID-19 on the Data Front and How to Reverse the Situation". JMIRx Med.

[3] Krukowski R (2021). Peer Review of "Why We Are Losing the War Against COVID-19 on the Data Front and How to Reverse the Situation". JMIRx Med.

[4] Anonymous (2021). Peer Review of "Why We Are Losing the War Against COVID-19 on the Data Front and How to Reverse the Situation". JMIRx Med.

[5] Anonymous (2021). Peer Review of "Why We Are Losing the War Against COVID-19 on the Data Front and How to Reverse the Situation". JMIRx Med.

